# The Effects of Combined Use of Sodium Citrate and PCE Plasticizer on Microstructure and Properties of Binary OPC-CAC Binder

**DOI:** 10.3390/ma17235901

**Published:** 2024-12-02

**Authors:** Victoria Shvetsova, Vadim Soloviev, Evgenii Matiushin, Vladimir Erofeev

**Affiliations:** Department of Building Materials Science, National Research Moscow State University of Civil Engineering (NRU MSUCE), Yaroslavskoe Shosse, 26, 129337 Moscow, Russia; solovevVG@mgsu.ru (V.S.); matyushinev@mgsu.ru (E.M.); erofeevvt@mgsu.ru (V.E.)

**Keywords:** sodium citrate, mixed binder, polycarboxylate ether plasticizer, mixed binder, environmental scanning electron microscope, X-ray diffraction, isothermal calorimetry, compressive strength

## Abstract

This study examines the impact of sodium citrate and a plasticizing additive, along with their sequential introduction into a cement slurry or concrete mix, on the heat evolution of the cement slurry, the microstructure, phase composition of the cement paste, and the compressive strength of fine-grained concrete. The binder used in this research was a blended binder consisting of 90% Portland cement and 10% calcium aluminate cement. This type of binder is characterized by an increased heat evolution and accelerated setting time. The addition of sodium citrate at 5% of the binder mass alters the phase composition of newly formed compounds by increasing the quantity of AFt and AFm phases. The presence of sodium citrate significantly delays the hydration process of tricalcium silicate by a factor of 3.3. Initially, it accelerates belite hydration by 31.6%, but subsequently slows it down, with a retardation of 43.4% observed at 28 days. During the hardening process, the hydration of tricalcium aluminate and tetracalcium aluminoferrite is accelerated throughout the hardening process, with the maximum acceleration occurring within the first 24 h. During the first 24 h of hydration, the dissolution rates of tricalcium aluminate and tetracalcium aluminoferrite were 40.7% and 75% faster, respectively. Sodium citrate enhances heat evolution during the initial 24 h by up to 4.3 times and reduces the induction period by up to 5 times. Furthermore, sodium citrate promotes early strength development during the initial curing period, enhancing compressive strength by up to 6.4 times compared to the reference composition.

## 1. Introduction

The exploration and study of additives that enable control over the strength development of cement-based concretes represent a significant focus of research for many scientists [1,2,3]. This interest is driven by the improvements in quality of life, economic growth in numerous countries worldwide, and, consequently, the rising pace and volume of building and infrastructure construction. Depending on the climatic characteristics of the construction region and the meteorological conditions specific to the building season, there is a demand for additives that either accelerate or retard the setting and hardening processes of Portland cement-based systems.

Accelerator additives are extensively utilized when an increase in the rate of concrete strength development is required. These applications include enhancing form turnover at precast concrete plants, optimizing the shotcrete process, facilitating the construction of buildings via 3D concrete printing technology, and reducing energy consumption during the autumn–winter construction period by minimizing the need for heating. Additionally, accelerators aid in maintaining freshly placed concrete in dry, hot climates by improving workability and setting time [4,5,6]. Notably, some of these additives, depending on their dosage, can also function as retarders. Sodium citrate is one such versatile additive that can serve dual roles depending on its concentration in the concrete mixture.

Citric acid (CA), whose sodium salt is sodium citrate, is primarily recognized as a setting and hardening retarder in cement systems and as a retarder and foaming agent in gypsum systems [7,8,9,10,11,12]. It extends setting times, reduces heat release, enhances rheological properties, and improves compressive strength in slag–alkaline, geopolymer, and magnesium oxychloride cement systems [13,14,15,16,17]. Research has demonstrated that citrate ions increase the solubility of slag and fly ash, leading to a denser cementitious matrix without significantly altering the composition of the hydration products [18,19]. In ettringite-based binders, such as low-sulfate gypsum (LSG), CA acts as a setting retarder at dosages of 1–2%, significantly increasing compressive strength by approximately 44%. It inhibits early ettringite formation while promoting monosulfate formation and the subsequent conversion of ettringite to calcium monosulfate. The degree of ettringite conversion is directly proportional to the CA content [20,21]. Moreover, CA prolongs workability by delaying initial ettringite formation; however, it substantially retards Portland cement hydration, resulting in decreased compressive strength, even at 28 days [22]. The quantity or precipitation rate of ettringite in mixed binders containing both Portland cement and calcium aluminate cement is not notably affected by CA, which negatively affected the development of the early strength of the cement paste. When incorporated at a concentration of 1 wt%, CA enhances the formation of portlandite. However, at higher concentrations, it hinders the dissolution of cement hydrates. Among the tested concentrations, a 1 wt% CA dosage was the only one that did not lead to a strength reduction between 14 and 28 days [23]. Smillie and Glasser [24] further observed that citric acid, at a concentration of 15.6 mmol/L, was almost entirely removed from the pore solution within the first hour of cement hydration.

It has been found that polycarboxylate ether (PCE)-based plasticizers are effective in enhancing the initial flowability of calcium sulfoaluminate (CSA) cements. However, PCEs quickly lose their dispersive effectiveness over time due to the extensive formation of hydrates that coat their side chains. The incorporation of CA in conjunction with PCE mitigates this rapid loss of PCE’s dispersive efficiency over time. CA has been shown to delay hydration primarily by inhibiting the dissolution of ye’elimite and anhydrite and the formation of ettringite and AFm phases. Consequently, the consumption of PCE by hydration products is reduced, which helps to maintain the dispersive efficacy of PCE over time. However, the initial flowability decreases due to competitive adsorption between CA and PCE. Due to its high charge density and mobility, calcium citrate quickly adsorbs onto the surface of cement grains, partially hindering the adsorption of PCE [25].

Sodium citrate (SC), a trisodium salt of citric acid, is commonly known as additive E331 and is considered entirely harmless for human consumption. It is widely used as a food additive for flavoring or preservation and in pharmacology as an anticoagulant [26,27,28,29,30]. Aqueous solutions of SC typically have a pH range of 7.5 to 8.5.

When used at concentrations of 0.02–0.4%, SC acts as a setting retarder for cement paste. Some researchers report reductions in heat release, cement paste workability, and compressive strength in cementitious systems [31,32,33,34,35]. Studies by Wynn-Jones G. et al. have shown that SC at 2–5% enhances pumpability, reduces setting times, and improves compressive strength of cement paste [36]. SC at a dosage of 0.15% has also been found to serve as an effective corrosion inhibitor for Portland cement-based concretes, with the time to 90% corrosion of reinforcing steel increasing from 4 to 8 months [37]. In carbonated reinforced slag–alkali systems, SC significantly alters the morphology and chemical composition of the steel’s passive film. By interacting with Fe^2+^ ions, it forms an adsorptive film on the steel rebar surface and reacts with cations in the carbonated solution of alkali-activated fly ash (AAFA), resulting in the precipitation of several compounds on the outer layer of the passive film. This interaction significantly mitigates and slows down corrosion caused by Cl^−^ ions [38].

It is suggested that citrates facilitate the dissolution of ferrite phases by promoting surface complexation and dissolution processes [13,18,39,40,41]. Citric acid and its salts are commonly used as cement hydration retarders, although at higher dosages, they can act as accelerators [42,43,44,45]. The addition of SC has been found to reduce the magnitude of the overall negative surface charge and increase the average enthalpy of hydration by 2.8 times with a 2% SC dosage. Portlandite content decreases by 38% at three days of curing as the SC dosage increases, although it stabilizes by 28 days. This is likely due to an increase in the degree of hydration of the aluminoferrite phase, coupled with a decrease in the hydration rates of alite, belite, and tricalcium aluminate [46]. Authors have also reported an increase in overall porosity at three days, reaching up to 24% compared to the control sample without additives; however, by 28 days, this porosity level decreases to align with that of the control composition [35].

Leonovich S.N. et al. investigated the effects of SC on the properties of calcium aluminate cement and proposed that SC increases the solubility of clinker minerals by binding Ca^2+^ ions into poorly soluble compounds. When introduced at dosages of 1–10% from the mass of binder, SC was found to enhance mix flowability by 2 to 2.3 times, delay the initial and final setting by 88–95% and 85–93%, respectively, and improve 28-day compressive strength by 66–253%. It also reduced water absorption by 2.2–85% [47,48]. To improve the efficiency of the additive, the authors suggested pre-drying 5.5 aqueous sodium citrate at a temperature of 200–250 °C for 3–4 h to remove adsorption-bound water [49].

The analysis of the research results indicates that sodium citrate (SC) is predominantly used at concentrations of up to 0.4% of the binder mass, primarily as a retarder for the setting and hardening of concrete, and less frequently as a corrosion inhibitor for steel reinforcement. At higher dosages (over 1% of the binder mass), it is applied in slag–alkali and geopolymer concretes, as well as in magnesium oxychloride-based systems.

Calcium aluminate cement is used as a component for mixed binders based on Portland cement [50,51,52]. It is most often used together with a sulfate-containing component (for example, gypsum or anhydrite) or without it. Ternary binders (OPC, CAC, calcium sulfate) are used in cases where control of shrinkage and rapid hardening kinetics are required. For example, in self-leveling mixtures or for repair work [53]. Mixed binders consisting of Portland cement and CAC have an increased content of the aluminate phase with an insufficient amount of the sulfate component [54,55]. Such a binder has an accelerated setting time and strength gain, increased heat generation, and can be widely used in the field of concreting at low temperatures and repair work [56,57,58,59].

The authors conducted experiments to select the optimal composition of the mixed binder, the amount of sodium citrate, and type and amount of plasticizer [58,59,60,61,62]. Based on the results obtained, this work uses a mixed binder containing 10% calcium aluminate cement and 90% Portland cement; sodium citrate is introduced in an amount of 5% of the binder weight and a plasticizer based on polycarboxylate esters is used in an amount of 0.5% of the binder weight.

The purpose of the study was to evaluate the effect of the complex additive and the sequence of introducing sodium citrate and PCE on the properties of the mixed binder. Concrete mixtures containing sodium citrate and a plasticizer can be used, for example, for repair work and concrete work in the winter with or without warming up measures. This is the type of work that requires a concrete mixture with high heat releasing, rapid setting, and strength. The introduction of additives in different sequences allows for control of the hydration process, and, consequently, the properties of the concrete mixture and concrete (setting time, durability, strength, etc.) [56,57,58]. A joint study [7] explored the combined effects of direct electric curing (DEC) and SC on concrete structure formation. It was found that DEC accelerates early-stage cement hydration and enhances AFt to AFm phase transformation, significantly impacting the porous structure. Conversely, SC can refine the pore structure or reduce the porosity of cement paste, positively influencing the physical and mechanical properties of concrete and enhancing structural durability.

## 2. Materials and Methods

### 2.1. Materials

#### 2.1.1. Ordinary Portland Cement (OPC)

**Figure 1:**
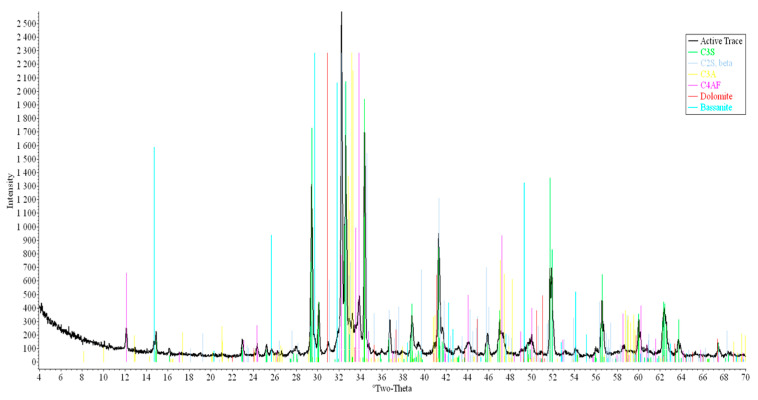
XRD pattern of Portland cement.

This study utilized gray Portland cement CEM I 52.5N, produced by Akkermann, Russia, with an alkali oxide content of 0.86% (expressed as Na_2_O equivalent). X-ray phase analysis was performed using an ARL X’TRA diffractometer (ARL, Lausanne, Switzerland), with measurement angles (2θ) ranging from 0° to +160°. The operational parameters included a tube voltage of 37 kV and a current of 35 mA. The resulting X-ray diffraction (XRD) pattern of the Portland cement is displayed in Figure 1, and its phase composition is detailed in Table 1.

#### 2.1.2. Calcium Aluminate Cement (CAC)

**Figure 2:**
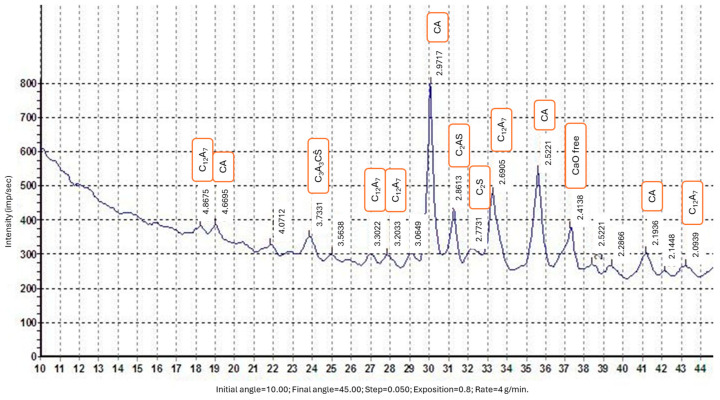
XRD pattern of calcium aluminate cement.

The study utilized calcium aluminate cement SRB400, produced by Kerneos, France. The X-ray diffraction (XRD) pattern of the calcium aluminate cement, shown in Figure 2, was obtained using a DRON-3M X-ray diffractometer (RPE Thunderbird, Saint Petersburg, Russia). The analysis was conducted at shooting angles of 2θ ranging from 10° to 50°, with a tube voltage of 30 kV and a current of 20 mA. The powdered sample was placed in quartz cuvettes, which rotated at an angular velocity of 1 revolution per second perpendicular to the radiation beam. The phase composition of the calcium aluminate cement is summarized in Table 2.

For qualitative phase analysis of cements, the ICDD PDF-2 2022 database was used. The analysis was carried out by interplanar distances in a semi-automated mode using the Crystallographica Search-Match software v3.1.0.2 with RDB support (Oxford Cryosystems Ltd., Oxford, UK). Non-standard quantitative X-ray phase analysis according to the Rietveld method was carried out using the Siroquant 4.0 (Sietronics Pty Ltd., Canberra, Australia) or ICDD PDF-2 2022 software v4.22.3.2 (ICDD, Newtown Square, USA).

Table 3 presents the results of tests conducted to determine the main characteristics of cements. The tests were carried out in accordance with GOST 30744-2001 [63].

#### 2.1.3. Sodium Citrate (SC)

Sodium citrate is the trisodium salt of citric acid Na_3_C_6_H_5_O_7_; is a crystalline white powder; molecular weight is 294.11 amu. The hydrogen index of a 1% aqueous solution is 7.5–9.0.

#### 2.1.4. Plasticizer Based on Polycarboxylate Ethers (PCE)

An additive based on polycarboxylate esters, Master Glenium 115 (MG115) (BASF), was utilized as a plasticizing additive. The density of the additive was ρ = 1.064 g/cm^3^ and pH = 5.04.

### 2.2. Test Methods

#### 2.2.1. Mix Proportions and Preparation of the Specimens

**Figure 3:**
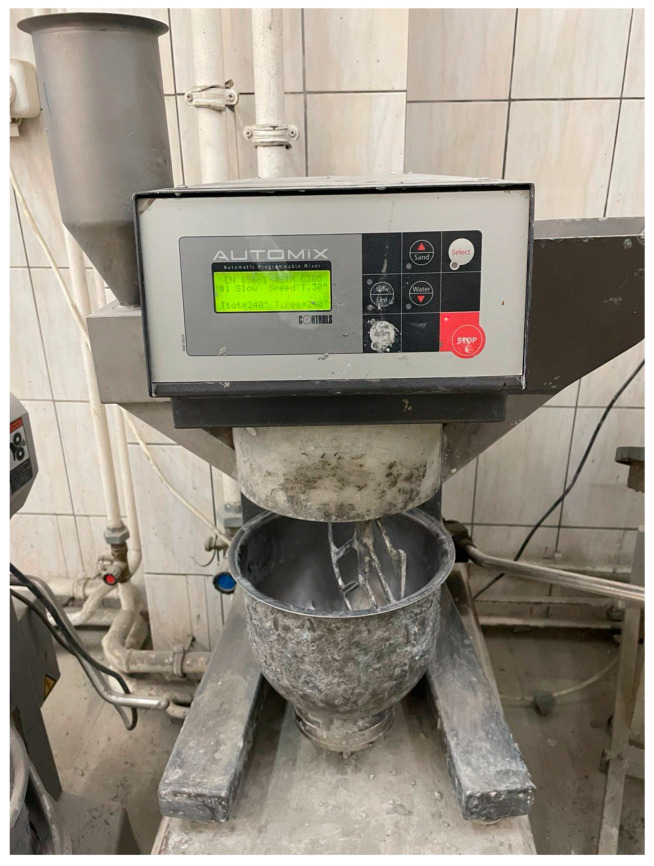
Mixer Controls.

Based on previously obtained results, it was determined to maintain the CAC content at 10% of the binder weight, the sodium citrate content at 5% of the binder weight, and the plasticizer content at 0.5% of the binder weight [58,59,60]. The compositions of the cement pastes are presented in Table 4. The sodium citrate and plasticizer were introduced in three distinct ways. The additive-free composition 1, consisting of 10% CAC and 90% Portland cement (hereinafter referred to as composition “10/90”), and the composition 2 without plasticizer, containing 10% CAC, 90% Portland cement, and 5% sodium citrate (hereinafter referred to as composition “10/90+SC 5%”), were used as references. The binder composition (10/90) and the additive quantities remained consistent throughout. The sequence of additive introduction is indicated in the labeling of the compositions and is elaborated upon in Table 5. Table 6 presents the formulations of fine-grained concrete from which samples were produced to evaluate compressive strength. The components were mixed in a mixer (Figure 3).

#### 2.2.2. Environmental Scanning Electron Microscopy (ESEM) Analysis

**Figure 4:**
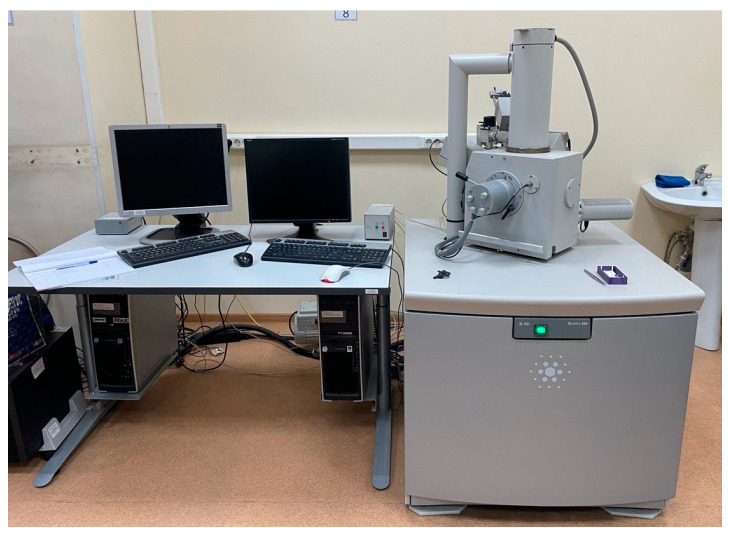
Microscope FEI Quanta 250.

To examine the hydration processes of the binder within the first 10, 30, and 55 min post-mixing, a scanning electron microscope (SEM) FEI Quanta 250 (FEI, Hillsboro, OR, USA) was employed (Figure 4). The cement paste was dozed using a pipette onto carbon tape glued to a table that was placed in the microscope chamber. Until the moment of shooting, the sample was kept in laboratory conditions. The tests were conducted in a natural vapor environment using a Peltier table, maintaining a reduced pressure between 900 and 650 Pa and a temperature of 2 °C within the microscope chamber.

#### 2.2.3. Isothermal Calorimetry

**Figure 5:**
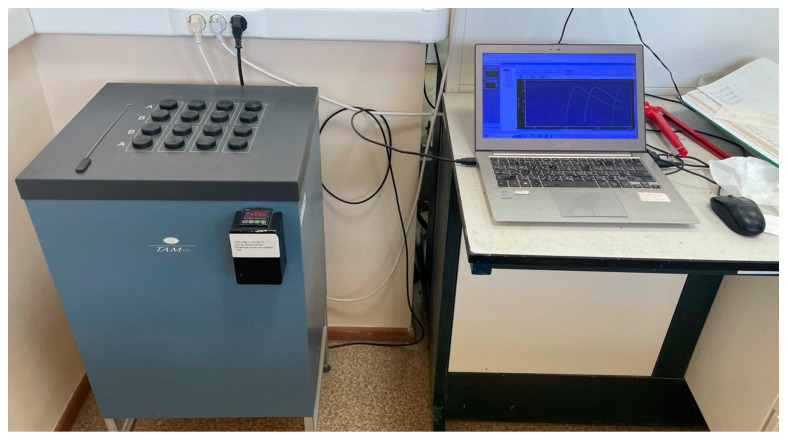
Isothermal calorimeter TAM Air.

The determination of the heat release of cement paste was carried out in accordance with ASTM C1702-17 [64]. The water–cement ratio in the pastes was 0.5; the nominal temperature in the thermostat was +20 °C. The studies of the heat release of cement paste were obtained using a TAM Air calorimeter (TA Instruments, New Castle, DE, USA) (Figure 5).

#### 2.2.4. XRD Analysis

**Figure 6:**
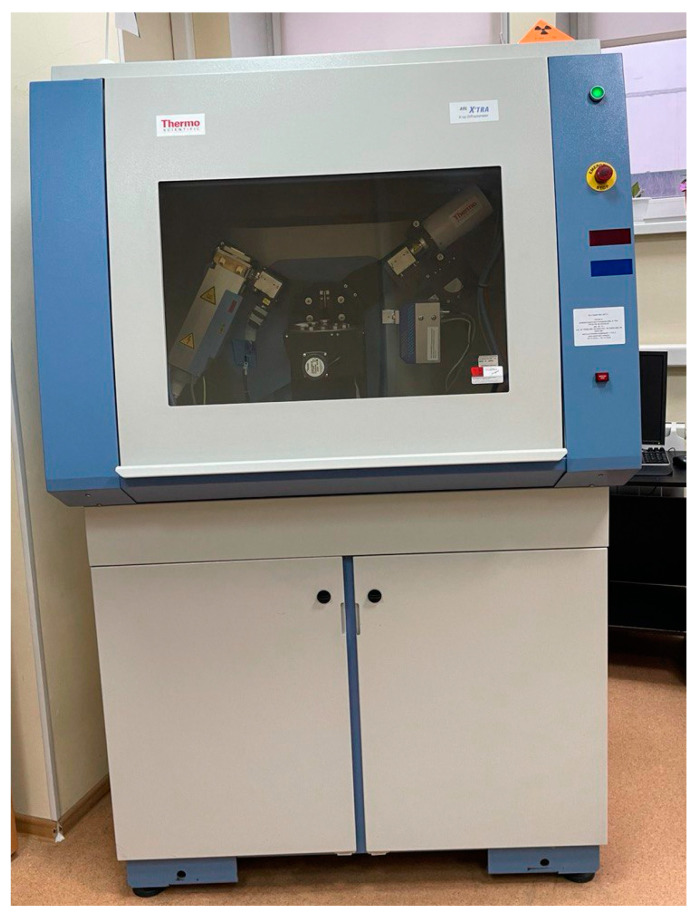
Diffractometer ARL X’TRA.

X-ray analysis of samples obtained from hardened cement paste was carried out using an ARL X’TRA X-ray diffractometer (ARL, Lausanne, Switzerland) equipped with an MS 61-04×12-long-focus tube (0.4 mm × 12 mm) CuKα, Ni filter (Figure 6).

#### 2.2.5. Compressive Strength

**Figure 7:**
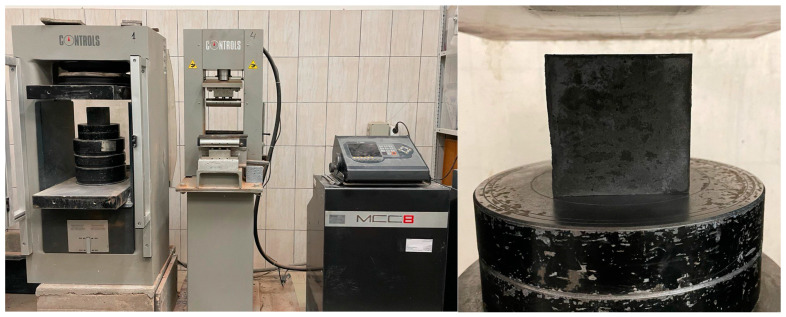
Testing machine Controls.

Fine-grained concrete samples with dimensions of 70 × 70 × 70 mm were prepared and subsequently tested at 1, 3, 12, and 28 days. Prior to testing, the samples were cured at a temperature of +21 ± 2 °C and a relative humidity of 90–100%. Following the curing period, the samples were subjected to compressive strength testing in accordance with GOST 10180-2012 [65] using a 50-C8422 MCC8 servohydraulic testing machine (Controls, Milan, Italy) (Figure 7).

## 3. Results

### 3.1. Effect of the Presence of Additives and the Sequence of the Addition of Sodium Citrate and Plasticizer on the Early Hydration of Cement Pastes

Table 7 presents the SEM images illustrating the microstructural evolution of cement pastes during the initial hour of hydration. These images, captured continuously starting 10 min after cement mixture preparation, were used to assess the influence of the sequential addition of sodium citrate (SC) and a polycarboxylate-based plasticizer on early-stage cement paste structure formation.

At 15 min after mixing, composition 1 (mixed binder without additives) displays distinct cement grains (Table 7, column a, row 1), with observable hydration products including cubic tricalcium hydroaluminate and hexagonal ettringite. In composition 2, which includes 5% of SC, cement grains are encased in a dense layer of prismatic hydration products approximately 2 μm in size (Table 7, column a, row 2). Notably, this composition exhibits high intergranular porosity that initiates soon after mixing. Composition 3, prepared with simultaneous addition of plasticizer and SC, shows cement grains bound by a gel-like hydration layer with a lower degree of crystallinity compared to composition 2 (Table 7, column a, row 3). When SC is added prior to the plasticizer, adsorption occurs on both cement grains and hydration products. The plasticizer’s steric effects inhibit the formation of crystalline intergrowths, thereby delaying the transition from a coagulation structure to a condensation–crystallization structure (Table 7, column a, row 4) [66,67]. In composition 5, where the plasticizer precedes SC addition, slow structure formation is evident, as plasticizer molecules adsorbed onto cement grains hinder hydration and interaction with SC, leaving binder grains without significant external hydration (Table 7, column a, row 5).

At 30 min after mixing, composition 1 exhibits well-formed hexagonal calcium monosulfoaluminate plates and cubic tricalcium hydroaluminate crystals (Table 7, column b, row 1), indicating an active hydration process [68]. Composition 2 shows prismatic ettringite crystals encased in a gel-like matrix, with visible ongoing intergranular void formation (Table 7, column b, row 2). Although the hydration of composition 3 initially lags behind composition 2, prismatic crystals emerge clearly by this point (Table 7, column b, row 3). In compositions 4 and 5, hydration proceeds at a reduced pace. Composition 4 displays spaced hexagonal ettringite crystals (Table 7, column b, row 4), while composition 5 contains minimal hydration products by 30 min (Table 7, column b, row 5), due to steric hindrance from the plasticizer.

At 55 min, composition 1 shows prominent calcium monosulfoaluminate plate-like crystals (Table 7, column c, row 1). Composition 2 retains numerous ettringite crystals with sizes between 1–2.2 μm (Table 7, column c, row 2) [69]. In composition 3, well-crystallized ettringite crystals are observed on cement grain surfaces (Table 7, column c, row 3). Hydration products in composition 4 form a cohesive but poorly crystallized system, with hydration lagging about 45 min compared to composition 2 (Table 7, column c, row 4). Composition 5, after 55 min, shows an increased presence of Portland cement hydration products, although their small size did not allow an assumption to be made about its composition (Table 7, column c, row 5).

The formation of a gel preceding the formation of AFm phases has been recorded by many researchers [69]. The gel is X-ray amorphous, since it consists of many thin plates growing on the surface of C_3_A. The conducted studies of the phase composition confirm the known assumptions that the aluminate and aluminoferrite phases hydrate with the formation of AFm and AFt phases, and after the exhaustion of sulfate ions, the Aft phase is replaced by the AFm phase. Subsequently, both phases tend to transform into hydrogarnet compounds. Also, Al(OH)_3_ and Fe(OH)_3_ are formed during the hydration of C_3_A and C_4_AF. The study of samples using X-ray diffraction did not allow them to detect these compounds, probably due to their weak crystallization, and, as a result, high X-ray amorphism.

### 3.2. The Influence of the Presence of Additives and the Sequence of Introduction of Sodium Citrate and Plasticizer on the Heat Release of Cement Paste

**Figure 8:**
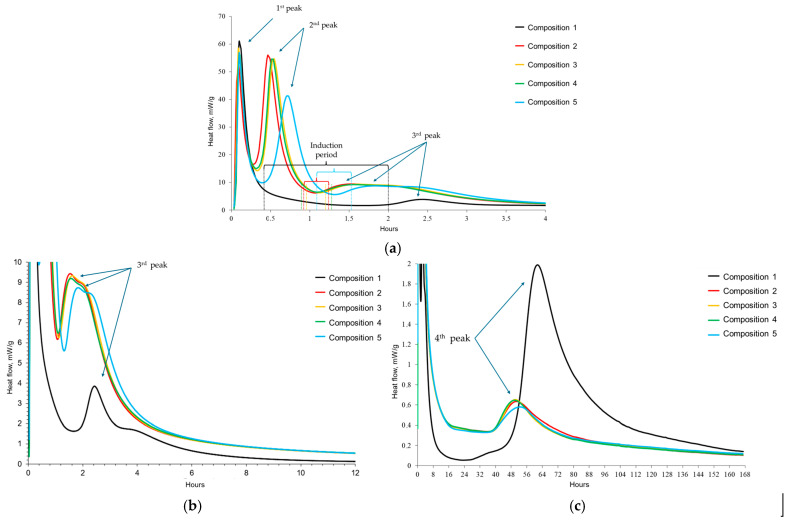
Dependence of heat flow on time: (**a**) 4 h; (**b**) 12 h; (**c**) 168 h.

The rate of heat evolution of different cement pastes is illustrated in Figure 8. The initial peak of the heat flow curve corresponds to the wetting of the cement, primarily involving the interaction between tricalcium silicate and calcium aluminates with water in compositions without additives, and tetracalcium aluminoferrite and tricalcium aluminate in compositions containing SC, as well as the dissolution of calcium sulfate hemihydrate [68]. Composition 1 displayed the highest intensity at this initial peak. In all compositions with SC, a second peak is observed between 28 and 43 min after paste preparation. Combined with SEM data, this peak likely signifies the formation of ettringite and calcium monosulfoaluminate phases.

The third peak for the control sample occurs after 2.6 h and characterizes the end of the induction period, which lasted about 1.5 h. The saddle-shaped peaks suggest two concurrent processes: ongoing hydration of clinker phases and the formation of new crystalline structures. Compositions containing SC, however, exhibit a markedly shortened induction period, lasting approximately 18 to 25 min (Figure 8a). For SC-containing compositions, the induction period begins approximately 30 to 40 min later than in the control sample, likely being delayed due to the active crystallization of the AFt phase.

The fourth peak is likely attributed to the depletion of calcium ions in the solution, originating from the hydrolysis of calcium aluminates—primarily calcium monoaluminate from CAC—and from the aluminate and aluminoferrite phases of Portland cement, as well as the reactivation of the hydration process of the silicate phases within Portland cement (Figure 8b). This peak is notably diminished in compositions containing sodium citrate, suggesting that SC inhibits the dissolution of clinker phases such as alite and belite. Key observations from Figure 8 are summarized in Table 8.

The adsorption of SC and PCE molecules on cement particles is competitive, with SC demonstrating a preferential adsorption advantage over PCE [70]. This preferential adsorption slows down the PCE adsorption rate, leading to considerable changes in the zeta potential of the paste, potentially even reversing it. This affects the dispersion of particles and subsequently alters the rheological properties of fresh pastes significantly [71]. Singh et al. [72] observed that increasing citric acid content decreases the zeta potential of cement, attributed to citrate ion adsorption on the positively charged surfaces of Portland cement grains. At low dosages, sodium citrate does not significantly alter calcium ion concentration in solution. Therefore, its retardation effect is primarily due to adsorption on mineral surfaces or hydration products, which inhibits mineral dissolution and C-S-H nucleation sites. However, at higher dosages, sodium citrate increases the initial calcium ion concentration in solution, forming complexes with calcium ions that further slowdown cement hydration. However, the initial concentration of calcium ions in solution increases at a high dosage of SC and complexation between citrate and calcium ions occurs, which also results in retarded hydration of cement [68].

**Figure 9:**
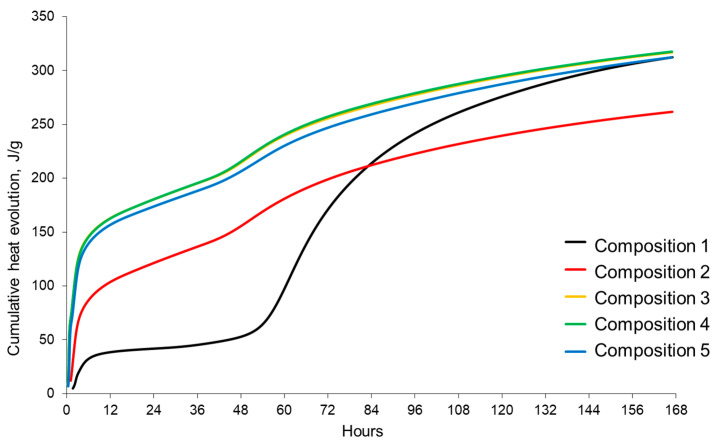
Cumulative heat evolution.

Despite a clear slowdown in the hydration processes and a decrease in their intensity for composition 5, the total heat release of this composition corresponds to a similar indicator for compositions 3 and 4. SC without a plasticizer (composition 2) accelerates hardening in the first 83 h, and composition 5 for 168 h. For compositions 3 and 4 with a plasticizer, the total heat release exceeds a similar indicator for the control composition and the composition with SC over the entire time interval of the study (Figure 9).

Among the primary clinker phases of the mixed binder (C_3_S, C_2_S, C_3_A, C_4_AF, CA), C_3_A and C_4_AF exhibit the highest enthalpy of solution. Upon the dissolution of these phases, ettringite and calcium monosulfoaluminate are formed, exhibiting the highest enthalpies of formation among the new formations and releasing the greatest amount of heat (Table 9). The above-mentioned processes of dissolution and crystallization, occurring in parallel, are accompanied by large emissions of thermal energy.

### 3.3. The Influence of the Presence of Additives and the Sequence of Introduction of Sodium Citrate and Plasticizer on the Phase Composition of Hardened Cement Paste

**Figure 10:**
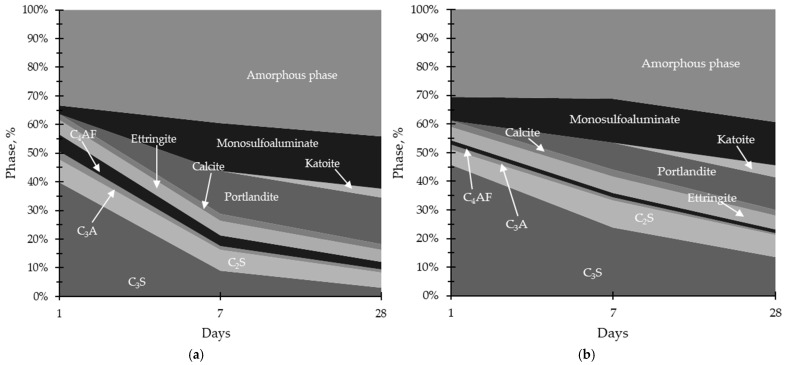
Mass fractions of phases of the composition: (**a**) 10/90; (**b**) 10/90+SC 5%.

XRD analysis was conducted on six samples, comprising three samples with a 10/90 composition and three samples with a 10/90+5% SC composition, evaluated at intervals of 1, 7, and 28 days. Qualitative phase analysis was performed using the Rietveld refinement method, facilitated by SIROQUANT software (v4.0). The resulting data are summarized in Figure 10.

Table 10 shows a comparison of the quantity of phases of the non-additive composition and the composition with sodium citrate. The addition of SC significantly reduced alite hydration, showing a linear slowdown that resulted in a 3.3-fold decrease by day 28 compared to the composition without the additive. Belite dissolution initially increased by 31.6% in the SC-containing samples but then slowed, with a 43.4% reduction by day 28. This initial increase in belite hydration could be due to the slowed alite hydration. In contrast, the hydration of tricalcium aluminate and tetracalcium aluminoferrite accelerated continuously, with peak acceleration occurring within the first 24 h. Specifically, in the SC-added samples on day 1, aluminate dissolution was 40.7% faster, and aluminoferrite dissolution increased by 75%. The significant heat release observed in the 5% SC compositions is likely linked to the high enthalpy of aluminoferrite dissolution (Table 9). According to Schwartz [74], citrate enhances the dissolution rate of ferrite phases, while in [32] it was found that citrate forms stable complexes with multivalent metal ions, affecting both the solution and surface chemistry of (ferro-)aluminates. The observed slow dissolution rates of alite and aluminate, along with the steady pore solution composition and rapid removal of citrate, suggest that citrate may precipitate or adsorb onto clinker grains. This likely forms a protective layer on the clinker surface, inhibiting clinker minerals dissolution [69]. Additionally, the hardening process involves substantial heat release, which is attributed to the formation and crystallization of ettringite.

However, ettringite is not the predominant phase formed. The main phase is calcium monosulfoaluminate, which includes partially substituted sulfate ions, hydroxide, and carbonate ions. In the presence of sodium citrate, monosulfate formation increases significantly, nearly doubling within the first day. This enhancement is due to the accelerated hydration of minerals in the presence of the SC, leading to early ettringite formation and quicker depletion of sulfate ions in solution, which promotes monosulfate formation. The detection of monosulfate by X-ray diffraction on day 1 indicates its early formation and good crystallization.

The reduction in portlandite content with SC addition is likely due to decreased alite solubility, as calcium ion supersaturation from aluminate and aluminoferrite phases occurs, along with the ettringite crystal growth on cement grains. Tricalcium hydroaluminate was only detected after 28 days, likely due to the recrystallization of metastable hexagonal calcium hydroaluminates (e.g., CAH_10_, C_2_AH_8_, etc.) [69]. These intermediate phases were not detected by XRD, suggesting C_3_AH_6_ crystallized directly from the solution. The recrystallization rate is influenced by factors such as temperature, pH, CO_2_ presence, water–cement ratio, and particle size [67]. Temperatures above 30 °C facilitate rapid transformations, and intermediate phases may not be observed due to local overheating from highly exothermic reactions [67].

A slight decrease in the amorphous phase content is also linked to slower hydration of alite and belite. No iron-containing compounds were detected by X-ray phase analysis, though some studies suggest complex citrate–iron compounds can form [38]. In this case, Fe(OH)_3_ formation is most likely, which, similar to Al(OH)_3_, could exist in an X-ray amorphous state.

### 3.4. Effect of the Sequence of Addition of Sodium Citrate and Plasticizer on the Compressive Strength of Concrete

**Figure 11:**
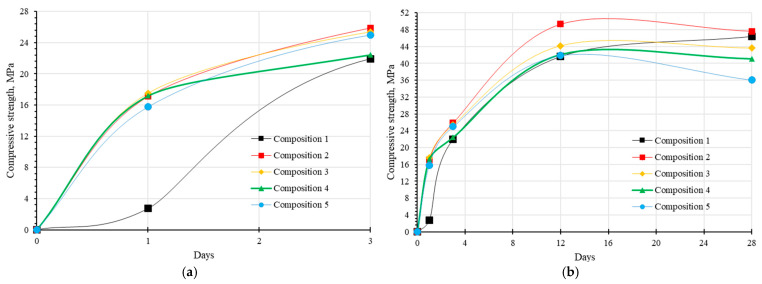
Dependence of concrete strength on the addition sequence of additives: (**a**) compressive strength of concrete samples at 3 days, (**b**) compressive strength of concrete samples at 28 days.

The compressive strength results are presented in Figure 11. On day 1, the control composition’s strength was five to six times lower than that of compositions with additives. This difference may be attributed to the formation of strong but small cubic hydrogarnet crystals [69]. Notably, the control composition exhibits slow hydration primarily during the first three days. By day 28, however, its compressive strength reaches levels comparable to the SC-containing compositions. After 12 days, a slight decrease in strength is observed for the samples with additives: composition 2 decreases by 3.4%, composition 3 by 1.2%, composition 4 by 2.4%, and composition 5 exhibits the most significant reduction, with a 14% drop. Composition 2, which lacks a plasticizer, consistently demonstrates the highest strength at all intermediate stages of hardening. Leonovich S.N. et al. found that adding 1–10% SC to cement compositions increased compressive strength by 66–253% after 28 days of hardening compared to compositions without additives [47]. The results obtained by Leonovich S.N. and others do not correspond to the results obtained in this work. This may be because a mixed binder was used in this work. By day 28, the compressive strength of composition 2 is slightly higher than that of the control composition, with an increase of approximately 2.7%.

The formation of the primary hydrate phase in Portland cement, C-S-H, is significantly delayed, which is typical for mixed cement systems [68]. In compositions with SC, the predominant phases are AFt and AFm phases. The accelerated hydration process in the presence of SC leads to the conversion of ettringite into monosulfoaluminate, which likely accounts for the high compressive strength observed from day one. Monosulfoaluminate crystals, which incorporate around 12 water molecules compared to the 26 water molecules in ettringite, exhibit a higher density and elastic modulus (as shown in Table 11), which can explain the higher compressive strength compared to control mixtures.

## 4. Conclusions

A study was conducted to investigate the sequence of introduction of sodium citrate and plasticizer for the heat release of cement paste, the microstructure and phase composition of hardened cement paste, and the compressive strength of fine-grained concrete prepared using a mixed binder, 10% calcium aluminate cement, and 90% Portland cement. Based on the findings, the following key conclusions were drawn:

1. Sodium citrate enhances the formation of both AFt (ettringite) and AFm (monosulfoaluminate) phases. Ettringite crystals, ranging in size from 1 to 2.2 μm, are distinctly observable after just 10 min of hydration in compositions 2 and 3 (with and without plasticizer, respectively). When sodium citrate and plasticizer are added separately, there is a noticeable delay in the development of the condensation–crystallization structure. This is attributed to competition between citrate ions and plasticizer molecules for adsorption sites on cement grains and hydration products. Due to their higher affinity, citrate ions preferentially adsorb onto the cement surface before polycarboxylate ether (PCE) molecules. Consequently, hydration is initially accelerated significantly, facilitating AFt phase formation. However, once the PCE molecules adsorb onto the AFt phase, they inhibit the establishment of inter-crystalline connections through steric repulsion, which substantially retards the structural development process.
2. The incorporation of sodium citrate to the binder significantly accelerates the heat release kinetics of cement pastes. Within the first 24 h of curing, the cumulative heat release in sodium citrate-containing compositions exceeds that of the control sample by a factor of 2.9 to 4.3. Sodium citrate notably reduces the induction period, with reductions of up to fivefold compared to the mixture without any additives. The sequence of additive incorporation also impacts the thermal response of cement pastes: adding a plasticizer prior to sodium citrate results in a modest reduction in heat release (approximately 3.7%).
3. The presence of sodium citrate markedly influences the hydration processes of individual clinker phases. Specifically, sodium citrate slows the hydration of tricalcium silicate, while it initially accelerates and then decelerates belite hydration. The hydration of tricalcium aluminate and tetracalcium aluminoferrite is consistently enhanced throughout the curing period, with the most pronounced acceleration observed within the initial 24 h. The primary hydration product, calcium monosulfoaluminate, is formed with partial substitution of sulfate ions by hydroxide and carbonate ions. In the presence of sodium citrate, the monosulfate content notably increases within the first day, reaching approximately 1.9 times the amount observed without the additive. The early detection of monosulfate within 24 h suggests robust crystallization and early phase formation.
4. Sodium citrate positively influences the early strength development of fine-grained concrete. On the first day, the control sample exhibited compressive strength values 5 to 6.4 times lower than those of the additive-containing formulations. Notably, composition 2—without plasticizer—demonstrated the highest strength throughout the first day of curing, likely due to the absence of plasticizer, which is known to decelerate cement hydration.
5. The substantial reduction in the hydration degree of alite and belite within the initial 28 days leads to an increase in the residual unreacted clinker phases. This preserved clinker content is expected to contribute to further strength gains and enhanced durability of concrete products and structures over prolonged service periods.

## Figures and Tables

**Table 1:** Mineralogical composition of Portland cement.

C_3_S	C_2_S	C_3_A	C_4_AF
63.3	18.0	5.6	9.5

**Table 2:** Mineralogical composition of calcium aluminate cement.

CA	C_12_A7	C_2_AS	C_2_S
85.0	3.0	5.0	3.0

**Table 3:** Properties of cements.

Type of Cement	Specific Surface, m^2^/g	Density, kg/m^3^	Consistency, %	Initial Setting Time, min	Final Setting Time, min	Compressive Strength 28 Days, MPa	Flexural Strength 28 Days, MPa
CEM I 52.5N	353	3158	31.25	134	192	50.9	6.2
SRB400	301	3216	26.75	110	279	60.3	6.4

**Table 4:** Mixture proportions for preparing cement pastes.

Name of Components	Mass Fraction of Components Relative to the Mass of Binder
Binder:	1
Ordinary Portland cement	0.9
Calcium aluminate cement	0.1
Water	0.5
Plasticizer	0.005
Sodium citrate	0.05

**Table 5:** Methods for preparing cement pastes.

Number of Mix	Name of Mix	Sequence of Additive Addition
1	10/90	Mix without additives (control).
2	10/90+SC 5%	Sodium citrate was mixed with water until completely dissolved; the resulting solution was used to mix the binder.
3	10/90+(SC 5%+MG115 0.5%)	Sodium citrate was dissolved in water, then a plasticizer was added to the water. The resulting solution is used to mix the binder.
4	10/90+SC 5%+MG115 0.5%	Sodium citrate was dissolved in water; the resulting solution was used to mix the binder. After stirred for 30–60 s, a plasticizer was added to the cement paste.
5	10/90+MG115 0.5%+SC 5%	The plasticizer was dissolved in 2/3 of water, the binder was mixed with the resulting solution, the paste was stirred for 30–60 s, then the remaining 1/3 of water with sodium citrate was added.

**Table 6:** Mixture proportions for preparing concrete.

Name of Components	Mass Fraction of Components Relative to the Mass of Binder
Binder:	1
Ordinary Portland cement	0.9
Calcium aluminate cement	0.1
Water	0.5
Sand	3
Plasticizer	0.005
Sodium citrate	0.05

**Table 7:** SEM patterns of cement pastes.

No.	15 min (a)	30 min (b)	55 min (c)
1	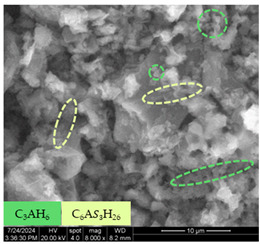	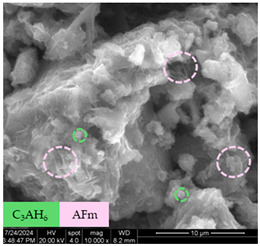	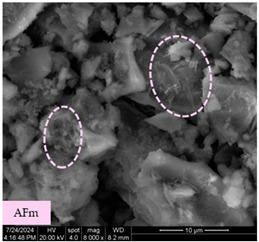
2	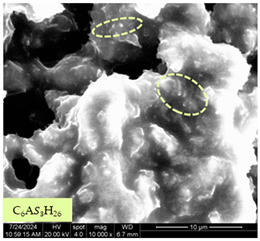	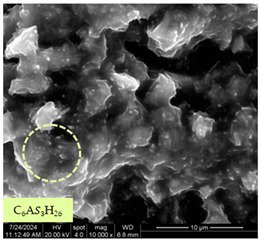	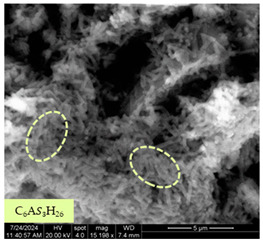
3	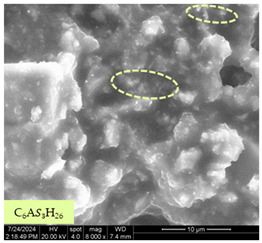	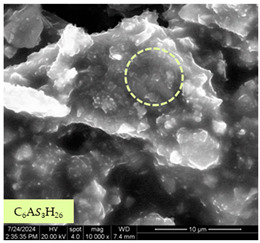	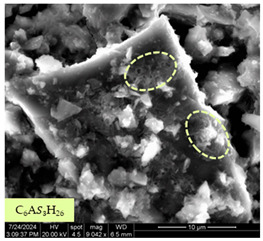
4	* 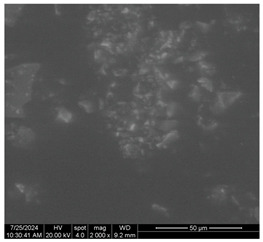 *	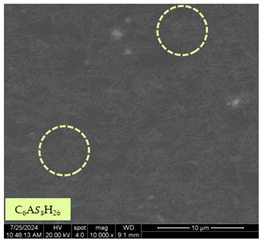	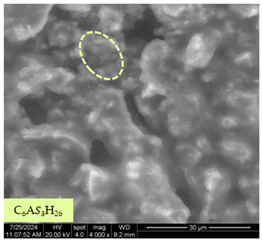
5	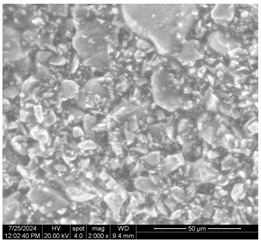	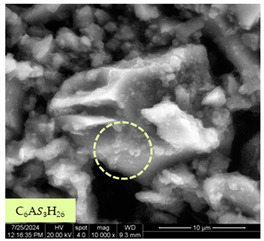	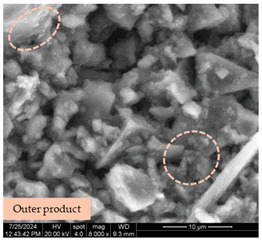

**Table 8:** Properties of peaks of heat release.

Number of Mix	1st Peak, min	2nd Peak, min	3rd Peak, min	4th Peak, h	Duration of the Induction Period, min	Cumulative Heat Evolution Through 24 h, J/g
1	0–6	-	160	62	90	41.8
2	0–6	28	76	51	18	121.3
3	0–6	31	80	50	18	180.4
4	0–6	33	76	49	18	180.5
5	0–6	43	100	53	25	173.8

**Table 9:** Enthalpy of dissolution of clinker phases and formation of new compounds.

Compound	ΔH^0^, kJ/mol	Reference
C_3_S	−2927.8	[73]
C_2_S	−2311.6	[73]
C_3_A	−3587.8	[68]
C_4_AF	−5090.3	[68]
CA	−2327.7	[69]
AFt	−17,539	[68]
AFm	−8752	[69]
CaCO_3_	−1207	[69]
Ca(OH)_2_	−986.1	[73]
C_3_AH_6_	−5548	[69]

**Table 10:** The difference in phase content between the composition without additives and the composition with 5% SC.

Name of Phase		Difference Between the Content of Phases with Non-Additive Composition, %		
		1 Day	7 Day	28 Day
Alite (C_3_S)	3CaO·SiO_2_	14.8	169.3	332.3
Belite (C_2_S)	2CaO·SiO_2_	−31.6	27.4	43.4
Tricalcium aluminate (C_3_A)	3CaO·Al_2_O_3_	−40.7	−14.3	−30.0
Tetracalcium aluminoferrite (C_4_AF)	4CaO·Al_2_O_3_·Fe_2_O_3_	−75.0	−61.1	−51.9
Ettringite	Ca_6_Al_2_(SO_4_)_3_(OH)_12_· ·26H_2_O	−4.2	11.8	17.1
Calcium monosulfoaluminate with partially substituted sulfate ions, hydroxide, and carbonate ions	3CaO·Al_2_O_3_·0.17CaSO_4_· ·0.17Ca(OH)_2_·0.66CaCO_3_·xH_2_O	189.7	−7.3	−18.0
Calcite	CaCO_3_	0.0	−16.7	−10.0
Portlandite	Ca(OH)_2_	-	−36.5	−30.2
Katoite, silicated	Ca_3_Al_2_(SiO_4_)_2_(OH)_4_	-	-	34.4
Amorphous phase		−9.1	−20.5	−11.4

**Table 11:** Properties of AFt and AFm phase.

Phase	Density, kg/m^3^	Reference	Modulus of Elasticity, MPa	Reference
Ettringite	1.78	[73]	22.4	[75]
Calcium monosulfoaluminate	1.99	[73]	42.3	[75]

## Data Availability

The data presented in this study are available on request from the corresponding author. The data are not publicly available due to privacy.
